# Touch Neurons Have a Good Sense of Direction

**DOI:** 10.1371/journal.pbio.1000304

**Published:** 2010-02-02

**Authors:** Richard Robinson

**Affiliations:** Freelance Science Writer, Sherborn, Massachusetts, United States of America

One of the most famous illustrations in all of biology is that of the somatosensory homunculus—a little man stretched along the brain's surface, each body part swollen or shrunken to match the size of the underlying neural real estate that receives its sensations from touch receptors in the skin. But while the illustration indicates where tactile sensation is recorded in the brain, it tells nothing about how. In a new study in this issue of *PLoS Biology*, Yu-Cheng Pei, Sliman Bensmaia, and colleagues address part of that deficiency and show that in at least one respect, the somatosensory system is like the visual system—it contains a class of direction-sensitive neurons that care not about what is moving along the skin, but only where it is going.

Incoming sensory signals from the skin travel up the spinal column, through the thalamus, and to the primary somatosensory cortex (called S1), a region on the surface of the brain just above and slightly behind the ear. Neuroanatomists divide S1 into four areas, called 1, 2, 3a, and 3b. Previous studies have shown that areas 1, 2, and 3b contain neurons that respond to the direction in which a tactile stimulus moves, but it has not been clear whether these neurons are sensitive to other aspects of touch, such as the shape of the object.

To explore this question, the authors recorded from single neurons in S1 of monkeys while stimulating their fingerpads with a grid of pins a centimeter square. Each of the 400 pins in the grid could be independently raised or lowered. The device allowed them to create shapes in contact with the skin, each pin analogous to a pixel in a visual monitor. By raising and lowering adjacent pins in quick succession, the shapes could be swept across the skin, creating the sensation of movement.

The authors asked how the neurons in S1 responded to the movement of three different shapes—a bar, a regular pattern of individual dots moving together, and a random array of dots moving either in random or in concert in one direction. They found that individual neurons in area 1 were “tuned” to specific directions of motion. For instance, a neuron might fire strongly when the movement proceeded from left to right along the fingertip, but responded weakly or not at all if the same shape moved from right to left. Importantly, these neurons conveyed the same information about the direction of movement, regardless of the “shape” of the moving object. Though some neurons in area 3b and 2 also conveyed information about direction of motion, the strength of their direction tuning was strongly dependent on the shape of the stimulus.

Within the visual system, neurons may be selective for direction of movement, orientation, or both. The authors found that, similarly, many somatosensory neurons displayed a dual sensitivity. By altering the orientation of the bar relative to its direction of movement, they found that about a third of the neurons were sensitive to direction alone, about 15% to orientation alone, and about a third to both. For the latter group, movement perpendicular to orientation triggered the strongest response.

The ability of these results to explain human motion sensitivity is still unknown. However, the degree of directional sensitivity of area 1 neurons matched the sensitivity of humans undergoing a “clockwise-counterclockwise” discrimination test. Thus, the involvement of this system in making judgments about directionality of tactile sensations is likely to be similar in humans.

The intriguing similarity between the visual and somatosensory processing of motion complements earlier studies showing how the two systems process shape information similarly. Visual processing has been the subject of extensive research, and results from that research have been used to develop fruitful new hypotheses about how the brain interprets touch.

**Pei Y-C, Hsiao SS, Craig JC, Bensmaia SJ (2010) Shape Invariant Coding of Motion Direction in Somatosensory Cortex. doi:10.1371/journal.pbio.1000305**

